# Supplementary material for: Dual mTORC1/mTORC2 Inhibition as a Host-Directed Therapeutic Target in Pathologically Distinct Mouse Models of Tuberculosis
Source: Antimicrob Agents Chemother. 2021 Jun 17;65(7):e00253-21. doi: 10.1128/AAC.00253-21 (PMC8373221; doi:10.1128/AAC.00253-21)

Supplemental Data

Supplemental Methods: Pharmacokinetics of CC214-2 in mice

Formulation: Suspensions of CC214-2 were prepared in aqueous 0.5% carboxymethyl cellulose and 0.25% Tween-80.

Pharmacokinetic study in mice: Female B6C3F1 mice were treated with a single oral dose of CC214-2 at 20 or 40 mg/kg (n=4 per dosing group), administered by gavage. Plasma samples were collected at 0.5, 2, 4, 8 and 24 h following administration. Compound concentrations in plasma were determined by LC-MS/MS. Area under the curve (AUC) was calculated by standard non-compartmental methods using Phoenix software (Pharsight). Data are reported in Table S1 as mean of dosing group ± standard deviation (SD).

Table S1. Plasma pharmacokinetic parameters after a single oral dose of CC214-2

	Mean (±SD) parameter value, by dose	
Parameter	20 mg/kg	40 mg/kg
C_{max} (μM)	5.4 ± 1.2	6.6 ± 2.0
t_{max} (h)	0.5 ± 0	0.5 ± 0
$AUC_{(0-\infty)}$ (μM·hr)	14 ± 3	34 ± 10

Table S2. Experimental scheme for efficacy evaluation in BALB/c mice

	Time point and number of mice to sacrifice						
Regimen	D-47	D0	W4	W8	W8+12	W16+12	Total
Untreated	4	6	5	5			20
Rapamycin$_{4q3d(M,W,F)}$			5	5			10
CC214-2$_{30}$			5	5			10
RHZE				5		15	20
RHZE+rapamycin				5		15	20
RHZE+CC214-2				5		15	20
BpaL			5		15		20
BPaL+rapamycin			5		15		20
BPaL+CC214-2			5		15		20
TOTAL	4	6	30	30	45	45	160

Table S3. Experimental scheme for efficacy evaluation in C3HeB/FeJ mice

	Time point and number of mice to sacrifice						
Regimen	D-48	D0	W4	W8	W12+12	W16+12	Total
Untreated	4	8		8			20
Rapamycin$_{4q3d(M,W,F)}$				8			8
CC214$_{30}$				8			8
RHZE				8		20	28
RHZE+rapamycin				8		20	28
RHZE+CC214-2				8		20	28
BpaL			8		20		28
BPaL+rapamycin			8		20		28
BPaL+CC214-2			8		20		28
TOTAL	4	8	24	48	60	60	204

Table S4. Efficacy evaluation in BALB/c mice

	Time point and lung CFU (Mean±SD)				Proportion Relapsing	
Regimen	D-47	D0	W4	W8	W8+12	W12+12
Untreated	1.32±0.15	5.66±0.32	5.30±0.31	5.57±0.47		
Rapamycin			5.59±0.65	8.36±0.33		
CC214-2			5.20±0.45	5.09±0.47		
RHZE				0.65±0.20		9/15
RHZE+rapamycin				0.62±0.39		13/14
RHZE+CC214-2				0.57±0.37		7/15
BPaL			1.79±0.31		7/15	
BPaL+rapamycin			1.90±0.36		15/15	
BPaL+CC214-2			1.74±0.32		7/15	

Table S5. Efficacy evaluation in C3HeB/FeJ mice

	Time point and lung CFU (Mean±SD)				Proportion Relapsing	
Regimen	D-48	D0	W4	W8	W11+12	W15+12
Untreated	1.36±0.06	7.92±1.31		8.60±0.12		
Rapamycin			9.16±0.18†	8 Died		
CC214-2				8.07±0.70		
RHZE				3.41±0.69		12/16
RHZE+rapamycin				2.76±0.79		10/15
RHZE+CC214-2				3.32±1.46		3/15
BPaL			4.67±1.22		3/15	
BPaL+rapamycin			3.88±1.05		3/4	
BPaL+CC214-2			3.84±1.46		1/12	

Fig. S1. Quantitative analysis of histopathology in BALB/c (B) and C3HeB/FeJ (K) mouse lungs. Quantitative comparisons of the absolute areas of inflammation (A) and necrosis (B), and their respective ratios relative to the total lung surface (C, D) and to each other (E) are presented for BALB/c (B) and C3HeB/FeJ (K) mice. Mice treated with rapamycin (Rapa, R) have red tone symbols and those treated with CC214-2 (CC, C) have blue tone symbols; untreated (UT) mice have black symbols. Lines represent median values.

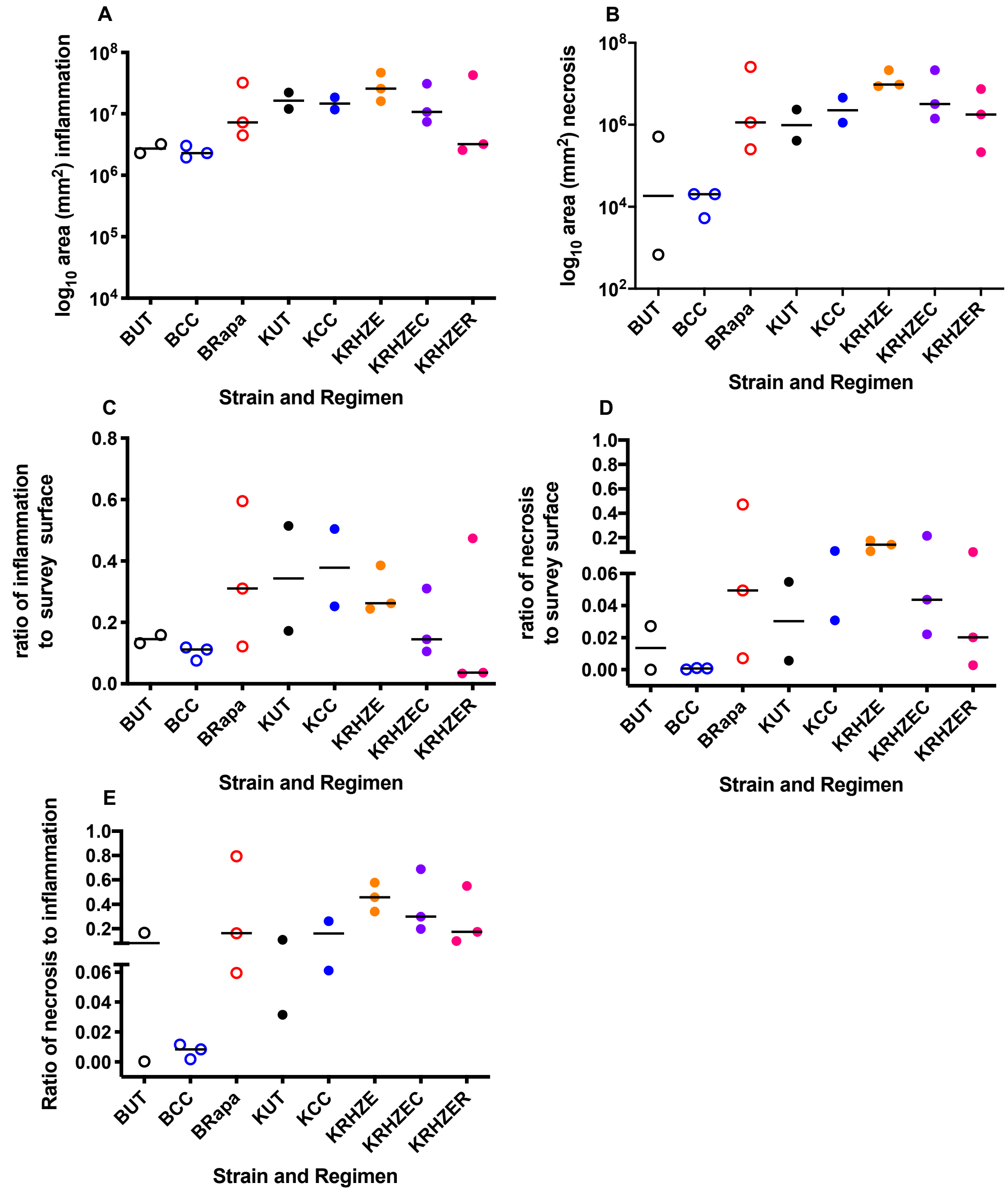

Supplement: Supplemental file 1 — Supplemental material. Download AAC.00253-21-s0001.pdf, PDF file, 0.2 MB [file aac.00253-21-s0001.pdf]
